# Ninety-day repeated oral toxicity study of saponified *Capsicum annum* fruit extract with 50% capsanthin in Sprague-Dawley Rats with a 28-day recovery period

**DOI:** 10.1016/j.toxrep.2022.03.007

**Published:** 2022-03-05

**Authors:** Velmurugan Shanmugham, Ravi Subban

**Affiliations:** Department of Chemistry, Karpagam Academy of Higher Education, Coimbatore 641021, India

**Keywords:** BW, Body weight, MCH, Mean Corpuscular Haemoglobin, ALB, Albumin, MCHC, Mean Corpuscular Haemoglobin Concentration, ALP, Alkaline phosphatase, MCV, Mean Corpuscular Volume, ALT, Alanine Aminotransferase, MON, Monocytes, APTT, Activated partial thromboplastin time, Na, Sodium, AST, Aspartate Aminotransferase, NEU, Neutrophils, BAS, Basophils, NOAEL, No Observed Adverse Effect Level, BUN, Blood Urea Nitrogen, PHO, Phosphate, Ca, Calcium, PLT, Platelet, Cl, Chloride, PT, Partial Thromboplastin time, CRE, CreatinineRBC, Erythrocyte count, EOS, Eosinophils, Retic, Reticulocytes, FBW, Fasting Body Weight, SD, Sprague-Dawley, GGT, Gamma Glutamyl Transpeptidase, T3, Triiodothyronine, GLOB, Globulin, T4, Thyroxine, Glu, Glucose, TBA, Total bile acids, HCT, Haematocrit, TBIL, Total Bilirubin, HDL, High Density Lipoprotein, TCHO, Total Cholesterol, HGB, Hemoglobin, TP, Total protein, K, Potassium, TRIG, Triglycerides, LDL, Low Density Lipoprotein, TSH, Thyroxine stimulating Hormone, LYM, Lymphocytes, WBC, Total leukocyte count, Saponified *Capsicum annum* fruit extract, *Capsicum annum*, Capsanthin 50% w/w, Toxicity, SD Rats, NOAEL, And Repeated dose

## Abstract

A ninety-day oral toxicity study of saponified *Capsicum annum* fruit extract with 50% (w/w) capsanthin (SCFE-50 C) was performed by oral gavage administration to male and female Sprague-Dawley (SD) rats at doses of 0, 500, 1000 and 2000 mg/kg BW/day for a period of ninety consecutive days. To assess the reversal of toxicity, the treatment phase was followed with a twenty-eight-day recovery period. The treatment with SCFE-50 C in both male and female SD rats showed no mortality, and no treatment-related toxicologically significant changes were observed in any groups. No significant differences between treated and control groups were found in feed consumption, body weight gain, individual organ weights, ocular examination, clinical chemistry or blood biochemistry. The necroscopy and histopathology examination did not reveal any clinically significant changes in male and female rats from the 2000 mg/kg BW/day group. According to this study, the no observable adverse effect level (NOAEL) for saponified *Capsicum annum* fruit extract with 50% (w/w) capsanthin (SCFE-50 C) administered by oral gavage for 90-days is > 2000 mg/kg BW/day in SD rats.

## Highlights

• Determination of no-observed-adverse-effect-level (NOAEL) of saponified *Capsicum annum* fruit extract with 50% (w/w) capsanthin (SCFE-50 C).
• The no-observed-adverse-effect-level (NOAEL) of the extract of SCFE-50 C in Sprague-Dawley rats was > 2000 mg/kg BW/day.
• No histopathological changes and no treatment-related adverse effects were observed in treated and control groups.
• *Capsicum annum* fruit extract with 50% (w/w) capsanthin (SCFE-50 C) is safe and well tolerated.

## 1 Introduction

Capsanthin (CAS 462–42–9) is a pepper-derived, lipophilic carotenoid, mostly found in the pericarp of ripe red pepper (capsicum spp.) and that is synthesized during carotenogenesis [1]. Capsanthin ((3 *R*,3’*S*,5’*R*)‐3,3’‐dihydroxybeta,kappa‐caroten‐6’‐one) has the molecular formula C_40_H_56_O_3_ and the molecular weight of 584.87 g/mol [2]. Capsanthin, a biological antioxidant, is a major carotenoid found in the red pepper to a maximum amount of 60% (w/w) of the total carotenoids [3]. Biological antioxidants can be classified into two major groups such as preventive and chain-breaking antioxidants. The role of preventive antioxidants is to suppress the chain-initiating radicals and thereby reducing lipid peroxidation whereas the chain-breaking antioxidants inhibit the formation of peroxide radicals [4]. Carotenoids can act as both preventive as well as chain-breaking antioxidants. In the preventive action, it quenches singlet oxygen and in chain-breaking, it suppresses the lipid peroxidation [5]. The phospholipids can be protected from oxidative damage by the chain-breaking action of carotenoids [6]. Capsanthin and capsanthin enriched paprika extract potentially inhibits colon carcinogenesis [7]. In a carbon tetrachloride-induced hepatotoxicity model, capsanthin conserved significant level of superoxide dismutase in carbon tetrachloride treated rats [8].

Capsanthin reduces the formation of malondialdehyde (MDA), which is one of the most known secondary products of lipid peroxidation and is used as a cell membrane injury marker [9]. In a Cu2 + catalyzed oxidation study, the LDL oxidation, and formation of dienes were suppressed by capsanthin. It also lowers LDL fraction and inhibits the auto-oxidation of cholesterol [10]. Saponified *Capsicum annum* fruit extract is widely used in processed food as an additive [11] and in dietary supplements for Age-related macular diseases (ARMD). In a subchronic toxicity study, no adverse effect was observed in rats fed with the saponified paprika extract 10,000 mg/kg feed corresponds to mean dosage of test item 858 mg (41.4 mg total carotenoids)/kg BW/day in males and 879 mg (42.4 mg total carotenoids) /kg BW/day [11]. Low-observed-adverse-effect level (LOAEL) of a pungency-masked and sustained release formulation of capsaicinoids-rich *Capsicum annum* extract using galactomannan, a soluble dietary fiber from fenugreek was determined for 500 mg/kg BW /day [12]. However, toxicological assessment on saponified extract standardized for > 50% (w/w) capsanthin and > 90% (w/w) total carotenoids has not been done and reported. It is, therefore, necessary to verify the toxicity effects of subchronic use of SCFE-50 C in rats to derive NOAEL. Accordingly, we tested the SCFE-50 C in rats to access the toxicity of its subchronic use.

## 2 Materials and methods

### 2.1 Test sample and reagents

#### 2.1.1 Vehicle control and Reagents

Vehicle control corn oil (Cat No. C8267) was procured from Merck (Merck Life Sciences Pvt Ltd, Bangalore, India). n-hexane (CAS 110–54–3, Cat No. 01082), ethyl acetate (CAS 141–78–6, Cat No. 010520) were procured from Spectrochem (Spectrochem Private Limited, Mumbai, India). Potassium hydroxide (CAS 1310–58–3, Cat No. 221473) was procured from Merck (Merck Life Science Pvt Ltd, Bangalore India). Capsanthin reference standard (Cat No. 19081, Lot# BCCC6238) was procured from Sigma-Aldrich (Sigma-Aldrich, Inc., St. Louis, USA). Tropicamide 1% Eye drops (Intas Pharmaceuticals, Mumbai, India) procured from a local pharmacy. Hematology analyzer calibration standard Minocal Calibrator (Cat No. 2032002, Lot No. 437) procured from Horiba (Horiba medical, Irvine, USA) and Clinical chemistry analyzer calibration standard Randox L2 (Human sera L2, Cat. No. HS2611, Lot No. 1221UN) procured from Randox (Randox Laboratories, London, England).

#### 2.1.2 Sample preparation

SCFE-50 C was prepared by extracting capsanthin ester from dried *Capsicum annum* fruits by solvent extraction method using n-hexane as a solvent. It was further purified by SCFE (Super Critical Fluid Extraction). The carotenoid esters were saponified by treating with 40% potassium hydroxide and purified by countercurrent extraction using ethyl acetate and water. The ethyl acetate layer was dried and concentrated under vacuum to obtain *Capsicum annum* extract which was standardized to contain > 50% (w/w) capsanthin and > 90% (w/w) total carotenoids [13] and complies with pre-determined specification as shown in Table 1. The SCFE-50 C was packed in light resistance low density polyethylene bags, purged with nitrogen, sealed and stored at 5 °C ± 3 °C.

**Table 1:** Specification of SCFE-50 C.

Parameter	Specification	Protocol
**Physical**		
Description	Dark red powder with characteristic odor	Organoleptic
Identification	To comply by HPLC and UV	HPLC and UV
Solubility	Soluble in organic solvents and insoluble in water	USP < 561 >
Loss on drying	Not more than 5.0%	USP < 732 >
**Chemical**		
**Assay**		
Capsanthin by HPLC	Not less than 50.0% and not more than 55.0% w/w	HPLC
Total Carotenoidsby UV.	Not less than 90.0% and not more than 95.0% w/w	UV
**Others**		
Lead	Not more than 5.0 ppm	ICP-OES
Arsenic	Not more than 3.0 ppm	ICP-OES
Cadmium	Not more than 1.0 ppm	ICP-OES
Mercury	Not more than 1.0 ppm	ICP-OES
**Microbiological profile**		
Total aerobic microbial count	Not more than 3000cfu/g	USP< 2021 >
Total yeast and mold count	Not more than 100cfu/g	USP< 2021 >
Escherichia coli	Negative/10 g	USP< 2022 >
Salmonella	Negative/10 g	USP< 2022 >
Staphylococcus aureus	Negative/10 g	USP< 2022 >
Pseudomonas aeruginosa	Negative/10 g	USP< 62 >

UV: Ultraviolet spectrophotometer, USP: United States Pharmacopeia, ICP-OES: Inductively coupled plasma-Optical emission spectroscopy, HPLC: High performance Liquid chromatography.

UV spectrophotometer: make-Shimadzu, Japan. Model-1900i. Instrument conditions: multiple wavelength scanning mode (190–800 nm).

HPLC: make-Shimadzu Japan, model: i-series plus LC-2030. Column-Sunfire L1 (Waters, USA). Dimension of 250 × 4.6 mm and particle size 5 µm. Mobile phase-binary gradient consists of acetone as solvent A and water as solvent B. Binary gradient program- 0–5 min, 75% B, 5–10 min, 75–95% B, 10–17 min, 95% B, 17–22 min, 95–100% B, 22–75 min, 100–75% B. The total flow rate-1.2 mL/min. Detection wavelength- 450 nm.

ICP-OES, make-Agilent, USA, Model-5510. Plasma conditions-Argon 15 L/min, Aux gas 0.8–1.0 L/min, analysis mode 500–700kpa. Nebulizer gas-0.7–1.2 L/min. RF power-700–1600 W

#### 2.1.3 Quantification of capsanthin in SCFE-50 C by HPLC

The quantification of capsanthin was carried out as per the method described in The Joint Expert committee on Food Additives (JECFA) capsanthin monograph [14]. Capsanthin was extracted using acetone, saponified and subjected to HPLC. Capsanthin reference standard from Sigma-Aldrich was used for quantification. Shimadzu HPLC, i-series plus LC-2030, (Shimadzu corporation, Kyoto, Japan) and Sunfire column L1 (Waters Corporation, Milford, USA) with a dimension of 250 × 4.6 mm and particle size 5 µm were used. The binary gradient consists of acetone as solvent A and water as solvent B and the binary gradient program were 0–5 min, 75% B, 5–10 min, 75–95% B, 10–17 min, 95% B, 17–22 min, 95–100% B, 22–75 min, 100–75% B. The total flow rate was set at 1.2 mL/min, and the detection wavelength was set at 450 nm. Capsanthin was identified in the samples based on the retention time of the capsanthin reference standard.

#### 2.1.4 Stability of SCFE-50 C

The storage stability study of SCFE-50 C at the storage condition 5 °C ± 3 °C was evaluated as per the International Conference on Harmonization guideline [15]. SCFE-50 C was filled in low density polyethylene bag (LDPE), purged with nitrogen and sealed. The LDPE bags was kept in stability chamber and temperature 5 °C ± 3 °C was maintained. The samples were analysed at the end of 0, 3, 6, 9 and 12 months. The parameters Description, Identification by HPLC, content of capsanthin and Total Carotenoids were analysed. The content of capsanthin was quantified by the HPLC method described in Section 2.1.2.

#### 2.1.5 Oxidative stability of SCFE-50 C extract in corn oil

The stability and homogeneity of the SCFE-50 C in corn oil were tested under study conditions. In this experiment, 5%, 10%, and 20% of SCFE-50 C in corn oil was prepared and stirred for three hours using a magnetic stirrer at room temperature. Samples were withdrawn at the interval of Initial, after 1, 2, and 3 h for analysis. The content of capsanthin was quantified by the HPLC method described in Section 2.1.2.

### 2.2 Regulatory guideline and ethics

This pre-clinical study was conducted by following OECD Guidelines for Testing of Chemicals, Section 4: Health Effects: No. 408, Repeated Dose 90-Day Oral Toxicity Study in Rodents; Adopted: 25th June 2018 [16]. OECD Principles on Good Laboratory Practice as revised in 1997 [17] were followed to conduct this study. This study protocol was approved by the Institutional Animal Ethics Committee (Approval No. VIP/IAEC/158/2019). This study was conducted at Vipragen Biosciences, a GLP accredited laboratory and certified by the National GLP Compliance Monitoring Authority (NGCMA) established by the Department of Science & Technology (DST), Government of India. The experiments were conducted by following all ethical practices as laid down in the guidelines for animal care and accredited by AAALAC international USA.

### 2.3 Animals

Sprague-Dawley (crl: CD (SD) male and female rats aged 5 weeks were procured from Hylasco Biotechnology (Ind) Pvt. Ltd., Hyderabad India (Charles River Laboratories USA, Technology Licensee) Male rats (n = 50) body weight ranged from 233.98 to 317.94 g and female rats (n = 50) body weight ranged from 158.56 to 223.38 g were used in the study.

The mean body weight (g) ± SD for G1, G2, G3, and G4 male rats are 271.67 g ± 23.33, 272.99 g ± 21.47, 275.11 g ± 24.42, and 274.93 g ± 17.14 respectively. Similarly, the mean body weight ± SD for G1, G2, G3, and G4 female rats are 183.5 g± 14.0, 186.9 g ± 13.02, 187.8 g ± 16.49, and 187.5 g ± 11.28.

The mean body weight for both male and female rats before commencing the study is well within the limit ± 20% as prescribed in OECD guideline 408-Repeated dose 90-day oral toxicity study in rodents [16]. All the animals were acclimatized for seven days after the veterinary examination.

Animals were housed under standard laboratory conditions such as adequate fresh air supply (Air changes 12–15 per hour), the range of room temperature and relative humidity were 19.6–24.9 °C, 48–66% respectively with light/dark cycle, 12 h each. In standard polycarbonate cages (Size: L 421 x B 290 x H 190 mm), two or three animals of the same-sex and group were housed. Facilities such as stainless-steel mesh for holding feed pellets and stainless-steel sipper tube for drinking water were provided in the cages. During the acclimatization and experimental period, the pellet feed (CRM (P) SQC, manufactured by SDS, UK) and reverse osmosis water were provided.

### 2.4 Vehicle and test item administration and observation

For vehicle control (G1) and vehicle control recovery (G1R) corn oil was used as such. Test item SCFE-50 C suspended at concentrations of 500, 1000 and 2000 mg/10mLin corn oil. The test item was prepared on daily basis and homogeneity was maintained using a magnetic stirrer. The actual dose volume for each animal was calculated based on the recent weekly body weight of the animals. Test items was prepared daily and the homogeneity was maintained using a magnetic stirrer. After the use, the SCFE-50 C packed in light resistance low density polyethylene bags, purged with nitrogen, sealed and stored at 5 °C ± 3 °C. For ninety consecutive days, the dose formulations (10 mL/kg/BW/day) were administered through an oral gavage route by using a 5 mL calibrated syringe with 0.2 mL scale graduation connected with gavage tube to the respective group rats once daily with the variation of ± 2 h from the first day. Similarly, vehicle (corn oil) at 10 mL/kg/BW/day was administered through oral route to rats in vehicle-control/vehicle control recovery groups for 90 consecutive days. Following the treatment period, the recovery groups were not given any dose formulation for 28 days. Every day, the animals were observed twice for mortality and morbidity during the trial period. This examination included cage-side, hand-held and open field observations that were recorded categorically. The clinical signs of toxicity were observed for all animals during the trial period and recorded on daily basis. The changes in eye pupil, fur, skin and mucous membranes were recorded. Besides, the autonomic activity such as respiratory pattern, piloerection and lacrimation, was also recorded. The bizarre behavior like walking backward & self-mutilation, clonic or tonic movements, and stereotypes such as repetitive circling and excessive grooming were also recorded. Since no treatment-related toxic sign/ functional deficit was observed during the experiment, functional examinations were not carried out for any of the main/ recovery group animals.

### 2.5 Ocular examination

Ocular examination was carried out for all rats on both eyes before start of treatment. At the end of the 90 days treatment period, the vehicle control and high dose group rat’s eyes were examined by a trained clinical veterinarian with an Ophthalmoscope. By using Tropicamide 1% solution, dilation was induced before the examination.

### 2.6 Feed consumption and Bodyweight gain

During the study period, the body weight was recorded once a week and at the end of the study period on the day of euthanasia before fasting. On weekly basis, feed consumption was measured during the dosing as well as the recovery period.

### 2.7 Urinalysis

Fresh urine was collected from all animals in a fasting state at the end of the study period and stored at 4ºC. Combur^10^ Test strips (Roche Diagnostic GmbH, Mannheim, Germany) used to measure Glucose, pH, and protein. Cobas u 411 urine analyzer instrument (Roche Diagnostics, Indianapolis, USA) was used to analyze urine chemistry parameters. The appearance and sedimentation were tested by visual inspection. Gravimeter (VET 360 Reichert Technologies, New York, USA) was used to check the specific gravity.

### 2.8 Hematology and blood chemistry

In this study, the hematology and blood chemistry parameters were selected based on the rat study reported by Tajri H et al. [18]. All the animals were kept in a biologic cage and blood samples were collected in the early morning between 7.00 and 9.00AM to reduce biological variation caused by circadian rhythms from all animals at the end of the experiment period under fasting conditions at least for 14 h but allowed access to water ad libitum. Under isoflurane anesthesia, blood was collected from the retro-orbital plexus [19]. Blood samples were collected for clinical biochemistry, hematology, and coagulation parameters. Blood samples for hematology analysis were collected in tubes previously filled with 10% K2-EDTA (dipotassium ethylene diamine tetra acetic acid) solution. For coagulation parameters, blood samples were collected in tubes previously filled with 3.2% sodium citrate. Plain tubes were used to collect the blood samples for clinical chemistry and centrifuged to collect the serum. For the estimation of serum total T4, T3, and TSH levels for all animals, additional blood samples were collected, centrifuged and the serum stored at − 70ºC. The hematological parameters [20] such as mean corpuscular haemoglobin (MCH), mean corpuscular volume (MCV), mean corpuscular haemoglobin concentration (MCHC), haematocrit (HCT), platelet count (PC), partial thromboplastin time (PT), activated partial thromboplastin time (APTT), total leukocyte count (WBC), hemoglobin (HGB), erythrocyte count (RBC) and reticulocytes (RETIC), were analyzed using Hematology Analyzer (ABX Micros ESV 60, Horiba UK Limited, Northampton, UK) which was calibrated by using calibration standard Minocal calibrator.

Giemsa stain was used to enumerate the cells and blood smear was prepared by using typical techniques and the cell count was expressed as percentage. Blood smear examination was conducted by using the differential count method and basophils (BAS), lymphocytes (LYM), neutrophils (NEU), monocytes (MOM) and eosinophils (EOS) were recorded. Clinical chemistry parameters such as glucose (GLU), total protein (TP), albumin (ALB), alanine aminotransferase (ALT), aspartate aminotransferase (AST), alkaline phosphatase (AP), total bile acids, globulin-calculated (GLOB), glucose (GLU), gamma glutamyl transpeptidase (GGT), total cholesterol (TCHO), high-density lipoprotein (HDL), low-density lipoprotein (LDL), creatinine (CRE), total bilirubin (TBIL), triglycerides (TRIG), blood urea nitrogen (BUN) - calculated, sodium (NA), calcium (CA), and chloride (CL) were estimated at the end of the study period [21] by using the instrument RX Daytona+ which was calibrated using calibration standard Randox L2.

### 2.9 Serum hormone analysis

2.9

Serum levels of thyroid hormones (THs) L-thyroxine (T4), L-triiodothyronine (T3) as well as thyroid-stimulating hormone (TSH) in control and treated groups were quantified. Concentrations of thyroid hormones in serum samples were analyzed by using enzyme-linked immunosorbent assay (ELISA) kits. T3 was calculated using a rat ELISA test kit (Cat No. K11–0535, Lot No. RT30720, KinesisDx, Brea, USA) and expressed in pg/mL, whilst T4 was calculated using a rat ELISA test kit (Cat No. K11–0338, Lot No. RT40720, KinesisDx, Brea, USA) and expressed in ng/mL. TSH was measured in IU/mL using a rat ELISA test kit (Cat No. K11–0181, Lot No. TSH0720, KinesisDx, Brea, USA).

### 2.10 Necropsy and histopathology

All rats were euthanized by CO_2_ asphyxiation followed by exsanguination. Rats were exposed to 20–30% CO_2_ in the euthanasia chamber until visible movements have ceased. The non-responsiveness of the animals was confirmed by the paw pinch reflex method. The animals were exsanguinated by severing the inferior vena cava after performing chest thoracotomy. A complete macroscopic postmortem examination [22] was performed on all rats, including the external surfaces of the carcass, all orifices, the cranial, thoracic, and abdominal cavities. The visible abnormalities were recorded. Organs such as heart, spleen, liver, brain, thymus, adrenal, kidney, epididymis or uterus, testes or ovaries, Prostate + seminal vesicles with coagulating glands, Thyroid gland (after fixation), and Pituitary gland (after fixation) were weighed and recorded. Organs and tissues were harvested and preserved in 10% neutral buffered formalin for necropsy analysis.

Organs and tissues such as the aorta, axillary/neck lymph node, adrenal glands, brain (cerebrum, cerebellum, medulla/pons), bone and bone marrow (femur), mesenteric lymph nodes, nerve, sciatic, spinal cord (cervical, thoracic and lumbar), skeletal muscle, skin (with mammary gland for male and female), cecum, epididymis, esophagus, trachea, rectum, prostate + seminal vesicles with coagulating glands, salivary glands, spleen, stomach, thyroid with parathyroids, thymus, duodenum, heart, colon gross lesions, Ileum with Peyer’s Patch, jejunum, kidneys, liver, lungs, ovaries, pancreas, pituitary gland, uterus with cervix vagina and urinary bladder. The eyes including the optic nerve and testes were harvested and stored in 10% neutral buffered formalin before that they were fixed in Davidson’s fixative.

For histopathological evaluation [22], specimens of the preserved tissues were embedded in paraffin and before that, they were trimmed and dehydrated. The paraffin-embedded tissues were stained with Mayer’s hematoxylin-eosin staining kit. A preserving medium 10% neutral buffered formalin was used to store all residual organs.

#### 2.10.1 Histopathology grading schemes

In histopathologic diagnosis, the severity grades are important and provide vital information about the treatment-related toxicity in the pre-clinical studies. The interstitial thickening and peribranchial inflammation were graded in parallel where 0 indicated no injury, grade 1 (minimal) indicated injury to 25% of the field, grade 2 (mild) indicated injury to 50% of the field, grade 3 (moderate) indicated injury to 75% of the field, and grade 4 (severe) indicated diffuse injury [23].

The severity of Hepatocellular necrosis graded as grade 0: within normal limits, grade 1 (minimal): approximately < 5% of centrilobular hepatocytes are necrotic, grade 2 (mild): approximately 5–20% of the liver is affected by centrilobular hepatocyte necrosis that is often circumferential, grade 3 (moderate): approximately 20–40% of the liver is affected by centrilobular hepatocyte necrosis that is often circumferential and bridging, grade 4 (marked): generally > 50% of the liver is affected by centrilobular hepatocyte necrosis that is bridging, confluent, and often extends beyond centrilobular zones [24].

Renal lesions in treated animals were assessed and graded into five categories by utilizing a scale of 0–5 [25]. Grade 0: normal histology, Grade 1 (minimal): tubular epithelial cell degeneration, without significant necrosis/apoptosis, grade 2 (mild): < 25%, grade 3 (moderate): < 50%, grade 4 (marked): < 75%, grade 5 (extreme): > 75% of the tubules showing tubular epithelial cell necrosis/apoptosis, respectively, accompanied by other concomitant alterations.

### 2.11 Analysis of capsanthin levels in macula and plasma

Eye and plasma were collected from the rats treated with SCFE-50 C for 90 days at different dose levels (G2, G3, and G4) and vehicle control (G1). Plasma from three rats from each groups was collected and mixed. Post sacrifice, the eye was collected, and the macula portion was excised. In each treatment group, macula from three rats was pooled and the homogenate was prepared using 500 µL of Phosphate buffered saline in presence of sterile metal beads. The homogenate was centrifuged (10000 rpm, 10 min at 4ºC) and the supernatant was flash-frozen using liquid nitrogen. Samples were stored in − 80ºC deep freezer until LCMS/MS analysis. Ethyl acetate 2 mL was added to 50 µL plasma or macula supernatant and vortexed for 10 min and clarified by centrifugation at 4000 rpm at 4 °C for 10 mins. From the supernatant, 1.5 mL was evaporated to dryness under nitrogen. The dried samples were reconstituted with 0.3 mL of mobile phase and injected into LCMS/MS. Waters LCMS/MS equipped with MassLynx V4.1 software was used and the instrument conditions were mode: positive ion spray, spray source: Quadrupole time of flight (QTof), Desolvation gas flow: 800 L/hr., desolvation temperature: 450ºC, Source temperature: 120ºC, capillary voltage: 2.5KV, cone:40 V, cone gas flow: 50 L/hr. The isocratic mobile phase consists of acetonitrile and 5 mM ammonium acetate (80:20 v/v) with 0.1% Formic acid and the flow rate was maintained at 0.7 mL/min. Atlantis®T3 column with a dimension of 4.6 × 75 mm, particle size 5 µm was used.

### 2.12 Statistical analysis

Graphpad Prism V7.04 [26] was used to perform Statistical analysis. Data on body weight, feed consumption, organ weights as well as clinical pathology data against the respective vehicle control group. Data normality was performed using Shapiro-Wilk test. Data for each group of animals were subjected to analysis of variance (ANOVA). T-test was used to compare the difference between control and treated recovery groups. Values were given as mean ± standard deviation (SD). All comparisons and analyses were evaluated at a 5% significance (P ≤ 0.05) level.

## 3 Results

### 3.1 Quantification of capsanthin

**Fig. 1:**
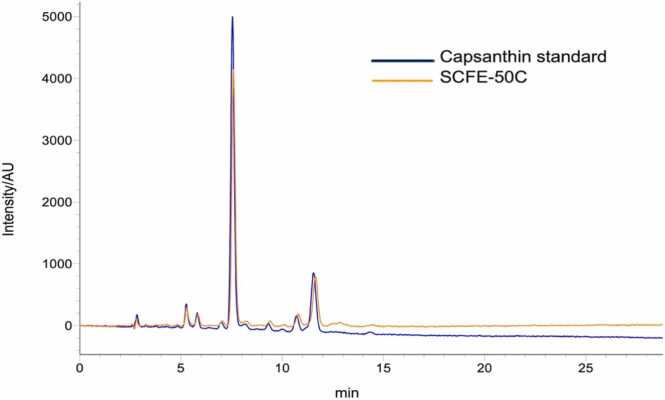
HPLC chromatogram of capsanthin standard and saponified *Capsicum annum* fruit extract with 50%w/w capsanthin (SCFE-50 C). Reference standard-capsanthin from Sigma-Aldrich (Cat No. 19081, LOT# BCCC6238). Instrument-Shimadzu HPLC, i-series plus LC-2030. Column-Sunfire column L1 (Waters, USA). Column dimension-250 × 4.6 mm and particle size 5 µm. The binary gradient consists of acetone as solvent A and water as solvent B and the binary gradient program were 0–5 min, 75% B, 5–10 min, 75–95% B, 10–17 min, 95% B, 17–22 min, 95–100% B, 22–75 min, 100–75% B. Detection wavelength-450 nm. Flow rate-1.2 mL/min. Capsanthin was identified in the SCFE-50 C based on the retention time of the capsanthin in reference standard.

Capsanthin was estimated in the Capsanthin SCFE-50 C according to the method described in Paprika extract, FAO JECFA monograph. Fig. 1 show HPLC chromatograms of capsanthin standard and SCFE-50 C. As shown in Fig. 1, the retention time of capsanthin in the standard solution was identical with capsanthin in the SCFE-50 C. A high abundance of capsanthin was eluted and resulting in capsanthin being the only major component present in capsanthin enriched extract. The content of capsanthin in SCFE-50 C was 50.59% (w/w) and the total carotenoids was 92.86% (w/w).

### 3.2 Stability of SCFE-50 C

The physical and chemical stability of SCFE-50 C was evaluated as per international conference on Harmonization guidelines. Test samples of SCFE-50 C was stored at 5º ± 3ºC for one year. The samples were packaged in Low density polyethylene bags and purged with nitrogen. Evaluations for physical and chemical stability were performed initially, after 3-, 6-, 9- and 12-months storage periods. Physical stability was assessed by organoleptic. The chemical stability of the SCFE-50 C was evaluated by means of a stability-indicating high-performance liquid chromatographic (HPLC) method. In addition, loss on drying and total carotenoids were also measured. At the end of one year, SCFE-50 C was stable at 5ºC. There are no physical and chemical changes observed at the end of the study period. The stability data are presented as Table 2.

**Table 2:** Stability of capsanthin content in the sample of saponified Capsicum annum fruit extract with capsanthin 50%w/w (SCFE-50 C) for the duration up to 12 months under the storage temperature 5 ± 3^º^ C.

Parameter	Specification	Initial	3 months	6 months	12 months
Description	Dark red pellets	Dark red powder	Same as initial	Same as initial	Same as initial
Identification	To comply by UV	Complies	Complies	Complies	Complies
Loss on drying	1.28%	1.42%	1.39%	1.56%	1.47%
Capsanthin	51.89%	51.78%	51.09%	50.88%	50.17%
Total carotenoids	92.86%	92.99%	92.32%	92.09%	91.58%
Change	–	No change	No change	No change	No change

A stability study of SCFE-50 C was performed as per ICH guidelines. Packets of 10 g each packed in light resistance Low-density poly bag, purged with nitrogen, and sealed. The samples of SCFE-50 C were kept at 5 ± 3º C for 12 months. Physical and chemical parameters were used to assess the stability. The samples were withdrawn and analyzed immediately at room temperature as per protocol (initial, 3, 6, and 12 months) and the data was compared with the initial values. The analytical data indicate that the physical, chemical parameters of SCFE-50 C remained within the specification during the stability period. The variation of the capsanthin content was found to be within the specified limit. The product is stable physically and chemically throughout the accelerated stability study.

UV spectrophotometer: make-Shimadzu, Japan. Model-1900i. Instrument conditions: multiple wavelength scanning mode(190–800 nm).

HPLC: make-Shimadzu Japan, model: i-series plus LC-2030. Column-Sunfire L1 (Waters, USA). Dimension of 250 × 4.6 mm and particle size 5 µm. Mobile phase-binary gradient consists of acetone as solvent A and water as solvent B. Binary gradient program- 0–5 min, 75% B, 5–10 min, 75–95% B, 10–17 min, 95% B, 17–22 min, 95–100% B, 22–75 min, 100–75% B. The total flow rate-1.2 mL/min. Detection wavelength- 450 nm.

### 3.3 Oxidative stability of capsanthin

The oxidative stability study of SCFE-50 C in corn oil revealed that Capsanthin was stable up to 3 h. No physical changes were observed in 5%, 10% and 20% of SCFE-50 C in corn oil throughout the study. The Capsanthin content was found to be within the specified limit for all the withdrawals and stable for 3 h at room temperature. The data are presented in Table 3.

**Table 3:** Stability of capsanthin content in the sample of saponified Capsicum annum fruit extract with capsanthin 50%w/w (SCFE-50 C) in the corn oil after magnetic stirring up to 3 h under the experimental temperature of 25º ± 2ºC using the HPLC analysis.

Period of keeping	Content of capsanthin by HPLC (% w/w)		
	5% in corn oil	10% in corn oil	20% in corn oil
Initial	5.42	10.86	20.93
After 1 h	5.31	10.71	20.70
After 2 h	5.38	10.69	20.52
After 3 h	5.26	10.43	20.29

A stability study of SCFE-50 C in corn oil was performed at room temperature (25º ± 2ºC). The 5%, 10%, and 20% w/w SCFE-50 C in corn oil prepared in an amber-colored beaker, covered with a lid, and stirred using a magnetic stirrer for up to 3 h at 25º ± 2ºC. The samples were withdrawn and analyzed immediately at room temperature as per protocol (after 1, 2, and 3 h) and the data was compared with the initial values. The analytical data indicate that the chemical parameters of SCFE-50 C remained within the specification during the stirring period. The variation of the capsanthin content was found to be within the specified limit. The product is stable physically and chemically throughout the study.

UV spectrophotometer: make-Shimadzu, Japan. Model-1900i. Instrument conditions: multiple wavelength scanning mode (190–800 nm).

HPLC: make-Shimadzu Japan, model: i-series plus LC-2030. Column-Sunfire L1 (Waters, USA). Dimension of 250 × 4.6 mm and particle size 5 µm. Mobile phase-binary gradient consists of acetone as solvent A and water as solvent B. Binary gradient program- 0–5 min, 75% B, 5–10 min, 75–95% B, 10–17 min, 95% B, 17–22 min, 95–100% B, 22–75 min, 100–75% B. The total flow rate-1.2 mL/min. Detection wavelength- 450 nm.

### 3.4 Clinical observation, body weight and feed consumption

**Fig. 2:**
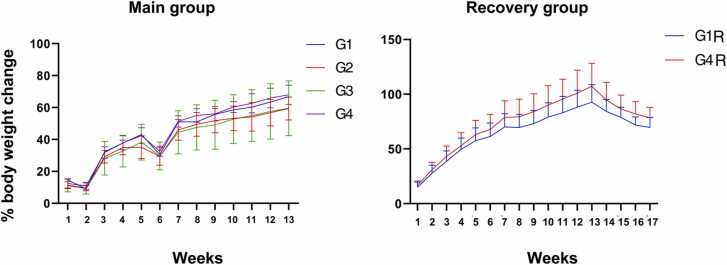
Summary of the male Sprague-Dawley rat% weekly body weight change after 90 days of oral repeated dose of SCFE-50 C, Values represent the mean ± SD (n = 10). No significant differences were observed.**,** G1: Vehicle control G2: Low dose SCFE-50 C, (500 mg/kg BW/day), G3: Mid dose SCFE-50 C, (1000 mg/kg BW/day), G4: High dose SCFE-50 C (2000 mg/kg BW/day).

**Fig. 3:**
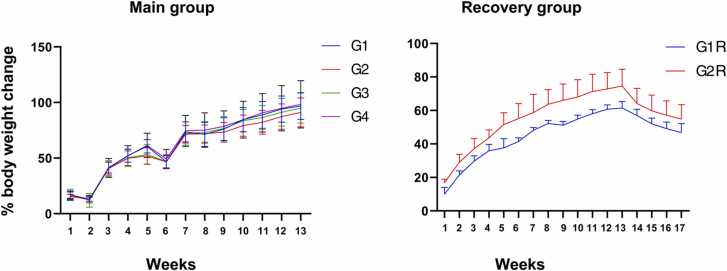
Summary of the female Sprague-Dawley rat% weekly body weight change after 90 days of oral repeated dose of SCFE-50 C, Values represent the mean ± SD (n = 10). No significant differences were observed.G1: Vehicle control G2: Low dose SCFE-50 C, (500 mg/kg BW/day), G3: Mid dose SCFE-50 C, extract, (1000 mg/kg BW/day), G4: High dose SCFE-50 C (2000 mg/kg BW/day).

**Fig. 4:**
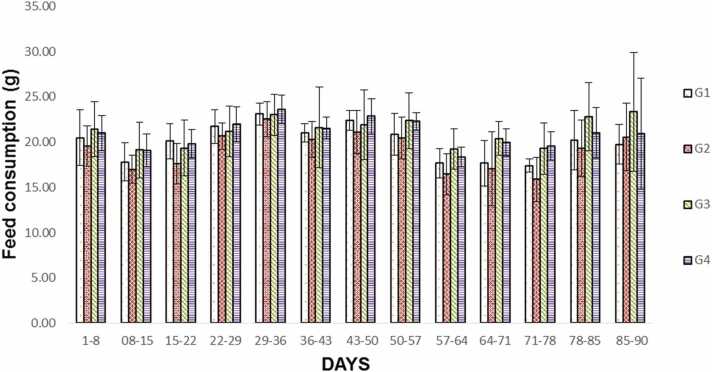
A Summary of the male Sprague-Dawley rats feed intake of 90 days of oral repeated dose of SCFE-50 C, Each point represents the mean ± SD (n = 10). No significant differences were observed, G1: Vehicle control G2: Low dose SCFE-50 C, (500 mg/kg BW/day), G3: Mid dose SCFE-50 C, (1000 mg/kg BW/day), G4: High dose SCFE-50 C (2000 mg/kg BW/day), Fig. 4B. Summary of the female Sprague-Dawley rats feed intake of 90 days of oral repeated dose of SCFE-50 C, Each point represents the mean ± SD (n = 10). No significant differences were observed, G1: Vehicle control G2: Low dose SCFE-50 C, (500 mg/kg BW/day), G3: Mid dose SCFE-50 C, (1000 mg/kg BW/day), G4: High dose SCFE-50 C (2000 mg/kg BW/day).

No mortality, abnormalities of body weight changes or food consumption were observed in the treated group in both sexes when compared to the respective control group. Statistically significant lower body weight gain during days 29–36 in G2 males (G1 = 22.49 g; G2 = 5.98 g) and statistically significant higher body weight gain during days 36–43 in G3 males (G1 = 8.18 g; G3 = 27.98 g) and lower body weight gain during days 29–36 in G2 females were observed (G1 = 9.65 g; G2 = 0.74 g). Statistically significant higher body weight gain during Days 1–8 (G1R=18.79 g; G4R=31.09 g), 29–36 (G1R=3.28 g; G4R=14.74 g) and 57–64 (G1R = −1.74 g; G4R =4.27 g) in females of G4R group. The weekly percentage mean body weight gain of the vehicle control and treated groups on week six was lower and over the course of the experiment was within the acceptable range. The percentage mean body weight change for main male and recovery group is given in Fig. 2. For female main and recovery groups are given in Fig. 3. Feed consumption was monitored from the first week for all groups G1 to G4 till the end of thirteen weeks (90 days) and was found to be comparable across all the test groups and the data are shown in Fig. 4A & 4B.

These changes in body weight gain were not dependent on the dose. Hence, these findings were considered incidental in nature. No treatment-related variation in the mean body weights and net body weight gains in any of the groups in both sexes when compared to the respective vehicle controls.

There were no treatment-related clinical signs observed in both sexes up to 2000 mg/kg BW/day. The clinical sign alopecia observed in animal No. 034, 035 of G2, 053 of G3, and 087 of G1R group females. These minor observations are not related to SCFE-50 C as they are not observed in any other groups. The ophthalmological examination did not reveal any abnormalities in all treated and control groups.

### 3.5 Hematology

The hematology analyzer was calibrated and found the results are agreeable with the calibration standard Toxicologically significant changes were not observed in all hematological parameters in all any groups and well within the historical control data.

In males, a statistically significant increase in eosinophil count was observed in G2 (1.8%) and in G3 (2.1%) when compared with the vehicle control group (1.0%). Females showed statistically significant decrease in WBC count in G4 (6.80 ×10^3^ cells/ mm^3^) (G1 =11.61 ×10^3^ cells/ mm^3^) and increase in level of platelet in G2 (722.20 ×10^3^ cells/ mm^3^), G3 (718.90 ×10^3^ cells/ mm^3^) and G4 (773.60 ×10^3^ cells/ mm^3^) groups in comparison to the vehicle control group (594.10 ×10^3^ cells/ mm^3^). However, the changes in the eosinophil count and platelet count in the main group were considered as treatment related but reversible after the treatment stopped.

In recovery males lymphocyte percentage (72%) (G1R=69%) was increased significantly. Coagulation parameters i.e., PT (15.02 s) (G1R= 14.19) was increased whereas, APTT (29.52 s) (G1R= 30.59) was decreased significantly when compared with the respective recovery control group. In recovery females, reticulocytes (3.0%) (G1R= 2.6%) and PT (14.16 s) (G1R= 14.10 s) were increased significantly in comparison to the recovery vehicle control group. In these parameters no corresponding changes were observed in the high dose group at the end of the treatment period. So, the changes observed were considered as incidental and toxicologically not relevant. The hematology parameters for male and female rats in all groups are shown in Table 4 and for recovery male and female groups are shown in Table 5.

**Table 4:** Summary of the male and female Sprague-Dawley rats hematological parameters after 90 days of oral repeated dose of SCFE-50 C.

	Males				Females			
	Control	SCFE-50 C (mg/kg BW/day)			Control	SCFE-50 C (mg/kg BW/day)		
Parameter		500	1000	2000		500	1000	2000
WBC (10^3^ cells/ mm^3^)	8.46 ± 2.67	8.86 ± 1.85	11.11 ± 2.79	10.16 ± 2.67	11.61 ± 1.62	9.40 ± 2.85	10.09 ± 2.37	6.80 * ± 3.36
RBC (10^6^ cells/ mm^3^)	8.65 ± 0.78	9.29 ± 0.86	9.16 ± 0.98	8.65 ± 0.38	8.05 ± 0.42	7.77 ± 0.25	7.91 ± 0.64	7.63 ± 0.60
HGB (g/dl)	15.01 ± 1.16	15.81 ± 1.43	16.06 ± 1.41	14. 98 ± 0.79	15.19 ± 0.62	14.71 ± 0.45	14.94 ± 0.72	14.45 ± 0.99
HCT (%)	46.02 ± 3.80	48.65 ± 4.69	49.23 ± 4.94	45.82 ± 2.54	45.17 ± 1.67	43.23 ± 1.19	44.13 ± 2.65	42.46 ± 3.41
PLT (10^3^ cells/ mm^3^))	745.90 ± 302.42	829.20 ± 183.46	706.40 ± 149.22	802.20 ± 90.78	594.10 ± 116.87	722.20 * ± 93.95	718.90 * ± 132.17	773.60 * ± 123.25
MCV (µm^3^)	53.40 ± 1.17	52.40 ± 1.26	53.80 ± 1.03	53.10 ± 1.79	56.30 ± 1.89	55.60 ± 1.51	55.90 ± 2.13	55.80 ± 1.14
MCH (pg)	17.38 ± 0.66	17.03 ± 0.38	17.58 ± 0.64	17.31 ± 0.48	18.89 ± 0.55	18.98 ± 0.52	18.96 ± 0.99	18.98 ± 0.57
MCHC (g/dl)	32.66 ± 0.83	32.54 ± 0.49	32.68 ± 1.02	32.68 ± 0.48	33.63 ± 0.76	34.08 ± 0.64	33.91 ± 0.88	34.12 ± 0.80
NEU (%)	24.10 ± 4.46	26.80 ± 2.39	25.90 ± 2.92	25.10 ± 2.28	25.50 ± 2.27	25.20 ± 2.70	24.90 ± 4.23	24.20 ± 4.47
LYM (%)	73.60 ± 4.27	70.10 ± 2.60	70.70 ± 2.50	71.90 ± 2.28	71.80 ± 2.30	71.60 ± 2.37	72.00 ± 4.24	72.00 ± 4.47
EOS (%)	1.00 ± 0.67	1.80 * ± 0.63	2.10 * ± 0.57	1.60 ± 0.70	1.30 ± 0.48	1.70 ± 0.82	1.70 ± 0.48	2.10 ± 0.88
MON (%)	1.30 ± 0.82	1.30 ± 0.48	1.30 ± 0.48	1.40 ± 0.52	1.40 ± 0.70	1.50 ± 0.53	1.40 ± 0.52	1.70 ± 0.48
BAS (%)	0.00 ± 0.00	0.00 ± 0.00	0.00 ± 0.00	0.00 ± 0.00	0.00 ± 0.00	0.00 ± 0.00	0.00 ± 0.00	0.00 ± 0.00
RETIC (%)	3.24 ± 0.30	3.14 ± 0.25	3.02 ± 0.48	3.05 ± 0.26	3.01 ± 0.71	2.93 ± 0.30	3.28 ± 0.36	2.62 ± 0.67
PT (second)	13.55 ± 2.60	14.86 ± 2.58	17.46 ± 7.35	11.68 ± 1.83	13.52 ± 4.15	11.93 ± 1.47	13.71 ± 3.51	13.59 ± 2.55
APTT (second)	37.41 ± 4.65	39.12 ± 3.62	35.70 ± 5.07	34.97 ± 6.54	39.18 ± 7.77	35.05 ± 5.22	36.29 ± 6.00	37.15 ± 6.89

N = 10. Values are in Mean ± SD *Significantly difference vs Control group (P < 0.05, Student’s t test).

WBC: Total leukocyte count, RBC: Erythrocyte count, HGB: Hemoglobin, HCT: Haematocrit PLT: Platelet, MCV: Mean Corpuscular Volume, MCH: Mean Corpuscular Haemoglobin, MCHC: Mean Corpuscular Haemoglobin Concentration, NEU: Neutrophils, LYM: Lymphocytes, EOS: Eosinophils, MON: Monocytes, BAS: Basophils, RETIC: Reticulocytes, PT: Partial Thromboplastin time, APTT: Activated partial thromboplastin time

**Table 5:** Summary of the recovery male and female Sprague-Dawley rats hematological parameters after 90 days of oral repeated dose of SCFE-50 C.

	Recovery males			Recovery females	
	Control	SCFE-50 C (mg/kg BW/day)		Control	SCFE-50 C (mg/kg BW/day)
Parameter		2000			2000
WBC (10^3^ cells/ mm^3^)	12.88 ± 3.38		13.56 ± 2.36	8.98 ± 1.10	9.68 ± 3.32
RBC (10^6^ cells/ mm^3^)	8.99 ± 0.65		8.51 ± 0.52	8.02 ± 0.50	7.82 ± 0.31
HGB (g/dl)	5.80 ± 0.65		15.40 ± 0.76	15.58 ± 0.75	14.86 ± 0.60
HCT (%)	47.38 ± 2.15		45.68 ± 2.58	45.62 ± 2.75	43.10 ± 1.96
PLT (10^3^ cells/ mm^3^))	638.20 ± 121.88		724.00 ± 140.95	687.40 ± 152.76	565.60 ± 225.27
MCV (µm^3^)	52.60 ± 1.82		53.80 ± 1.48	56.60 ± 0.89	55.00 ± 2.45
MCH (pg)	17.62 ± 0.76		18.14 ± 0.51	19.46 ± 0.53	19.04 ± 0.98
MCHC (g/dl)	33.36 ± 0.61		33.74 ± 0.58	34.18 ± 0.59	34.52 ± 1.14
NEU (%)	27 ± 2		26 ± 2	25 ± 4	24 ± 3
LYM (%)	69 ± 1		72 * ± 3	73 ± 4	73 ± 1
EOS (%)	2 ± 1		1 ± 1	1 ± 1	1 ± 1
MON (%)	2 ± 1		1 ± 1	1 ± 1	1 ± 1
BAS (%)	0.0 ± 0.0		0.0 ± 0.0	0 ± 0	0 ± 0
RETIC (%)	2.6 ± 0.4		2.8 ± 0.2	2.6 ± 0.3	3.0 * ± 0.2
PT (second)	14.19 ± 0.53		15.02 * ± 0.76	14.10 ± 0.76	14.16 * ± 1.70
APTT (second)	30.59 ± 1.09		29.52 * ± 0.80	31.08 ± 3.59	30.93 ± 0.66

N = 5. Values are in Mean ± SD *Significantly difference vs Control group (P < 0.05, Student’s t test).

WBC: Total leukocyte count, RBC: Erythrocyte count, HGB: Hemoglobin, HCT: Haematocrit PLT: Platelet, MCV: Mean Corpuscular Volume, MCH: Mean Corpuscular Haemoglobin, MCHC: Mean Corpuscular Haemoglobin Concentration, NEU: Neutrophils, LYM: Lymphocytes, EOS: Eosinophils, MON: Monocytes, BAS: Basophils, RETIC: Reticulocytes, PT: Partial Thromboplastin time, APTT: Activated partial thromboplastin time

### 3.6 Clinical chemistry

The clinical chemistry analyzer was calibrated before performing the analysis and found the results are well within the calibration standard specification. The Quality control report for clinical chemistry analyzer is shown in Table 7. In all clinical chemistry parameters, toxicologically significant changes were not observed in all any groups and complies with the historical control data. In males, a statistically significant increase in the level of creatinine was observed in G3 (0.63 mg/dL) in comparison with the vehicle control group (0.59 mg/dL). In females, the glucose level was increased statistically in G2 (87.00 mg/dL) and G4 (94.93 mg/dL) compared to control group (G1–64.92 mg/dL). However, no corresponding changes in these parameters were observed at the end of the recovery period. In recovery males, statistically significant decrease in level of GGT (−4.24 U/L) (G1R= −1.31 U/L), creatinine (0.71 mg/dL) (G1R=0.77 mg/dL) and chloride (103.00 mmol/L) (G1R=104.60 mmol/L) was observed when compared with the recovery vehicle control group. In female recovery group, total protein (68.46 g/L) (G1R= 67.52), globulin (45.88 g/L) (G1R= 44.42), sodium (132.42 mmol/L) (G1R= 130.82) and chloride (95.71 mmol/L) (G1R= 95.27 mmol/L level was increased. Whereas only Albumin (22.94 g/L) (G1R= 24.93) level was decreased in comparison to the respective control group. These treatment related minor changes observed in clinical chemistry parameters were not consistent in main and recovery groups and observed in only one of the sexes but reversed after treatment stopped. The summarized values of clinical parameters for male and female rats are shown in Table 6 and for recovery male and female groups are shown in Table 7.

**Table 6:** Summary of the male and female Sprague-Dawley rats clinical chemistry parameters after 90 days of oral repeated dose of SCFE-50 C.

	Males				Females			
	Control	SCFE-50 C (mg/kg BW/day)			Control	SCFE-50 C (mg/kg BW/day)		
		500	1000	2000		500	1000	2000
GLU (mg/dL)	83.59 ± 27.11	85.2 ± 27.57	94.19 ± 20.25	95.21 ± 17.2	64.92 ± 16.79	87.00 * ± 16.64	86.2 ± 21.33	94.93 * ± 25.86
UREA (mg/dL)	27.32 ± 2.1	26.78 ± 2.14	29.73 ± 3.75	27.58 ± 3.36	37.73 ± 6.37	34.93 ± 3.86	35.97 ± 4.36	32.51 ± 4.94
CRE (mg/dL)	0.59 ± 0.03	0.59 ± 0.02	0.63 * ± 0.04	0.62 ± 0.04	0.71 ± 0.06	0.72 ± 0.06	0.69 ± 0.04	0.69 ± 0.07
TCHO (mg/dL)	47.19 ± 5.64	51.33 ± 17.69	49.09 ± 10.2	50.29 ± 9.09	57.23 ± 11	57.44 ± 10.13	58.9 ± 13.29	59.75 ± 9.25
TRIG (mg/dL)	49.39 ± 15.12	41.22 ± 14.29	52.45 ± 18.56	56.47 ± 20.47	44.81 ± 15.54	43.8 ± 15.96	49.46 ± 17.62	46.94 ± 23.95
AST (U/L)	115.21 ± 33.46	125.28 ± 36.15	121.09 ± 31.58	99.87 ± 15.63	143.98 ± 65.6	141.99 ± 52.22	122.17 ± 25	131.21 ± 43.35
ALT (U/L)	40.07 ± 10.04	37.71 ± 7.73	43.67 ± 11.52	36.74 ± 5.27	40.45 ± 5.99	41.16 ± 8.09	33.59 ± 8.56	33.52 ± 6.6
TP (g/L)	64.99 ± 1.9	65.79 ± 3.03	68.66 ± 3.38	65.33 ± 4.33	76.75 ± 4.41	75.75 ± 4.81	73.51 ± 4.26	75.18 ± 2.49
HDL (mg/dL)	12.96 ± 1.91	15.98 ± 5.64	16.96 ± 3.66	17.31 ± 3.61	26.78 ± 5.59	30.63 ± 7.52	31.64 ± 6.8	32.52 ± 4.49
LDL (mg/dL)	9.1 ± 2.02	11.26 ± 6.6	11.95 ± 2.33	10.85 ± 4.34	17.52 ± 4.45	17 ± 6.21	18.72 ± 7.13	19.31 ± 4.03
ALB (g/L)	34.27 ± 8.4	34.63 ± 7.15	41.78 ± 11.3	31.57 ± 10.99	34.56 ± 7.28	33.11 ± 8.19	32.85 ± 5.71	36.5 ± 10.35
GGT (U/L)	0.4 ± 0.94	0.8 ± 1.33	0.22 ± 0.71	0.05 ± 0.16	-2.96 ± 2.8	-1.06 ± 5.96	-3.53 ± 2.03	-1.86 ± 5.86
CA (mmol/L)	2.69 ± 0.7	2.41 ± 0.34	2.2 ± 0.2	2.33 ± 0.55	2.63 ± 0.51	2.5 ± 0.53	2.38 ± 0.41	2.27 ± 0.3
PHO (mg/dL)	7.68 ± 0.89	7.7 ± 1.01	8.4 ± 1.95	8.04 ± 1.45	6.9 ± 1.34	6.69 ± 0.85	6.51 ± 0.92	6.67 ± 1.26
GLOB (g/L)	30.72 ± 7.05	28.67 ± 10.25	26.89 ± 13.72	33.76 ± 12.14	42.19 ± 8.28	41.63 ± 10.01	40.66 ± 8.27	38.68 ± 10.38
TBA (µmol/L)	25.23 ± 23.34	32.81 ± 34	12.53 ± 6.01	11.14 ± 5.5	34.41 ± 31.79	52.47 ± 53.31	50.42 ± 42.88	22.06 ± 12.51
ALP (U/L)	89.06 ± 31.16	69.45 ± 13.37	88.76 ± 41.9	117.64 ± 36.97	44.41 ± 21.89	41.58 ± 25.47	39.78 ± 23.06	47.46 ± 39.41
TBIL (mg/dL)	0.31 ± 0.04	0.37 ± 0.17	0.28 ± 0.07	0.31 ± 0.05	0.25 ± 0.08	0.31 ± 0.07	0.33 ± 0.07	0.29 ± 0.13
BUN (mg/dL)	12.77 ± 0.98	12.51 ± 1	13.89 ± 1.75	12.89 ± 1.57	17.63 ± 2.98	16.32 ± 1.8	16.81 ± 2.04	15.19 ± 2.31
NA (mmol/L)	143.2 ± 1.23	143 ± 1.63	143.8 ± 1.55	143.3 ± 1.7	144.7 ± 1.95	142.7 ± 1.16	143.6 ± 2.01	144.4 ± 1.78
K (mmol/L)	5.68 ± 0.63	5.58 ± 0.84	5.43 ± 0.58	5.11 ± 0.54	5.3 ± 0.88	4.75 ± 0.54	4.98 ± 0.73	4.62 ± 0.38
CL (mmol/L)	104.4 ± 1.43	104.8 ± 1.55	105.3 ± 1.06	104.6 ± 1.51	105.1 ± 1.66	102.3 ± 1.34	104.7 ± 1.49	105.9 ± 1.79

N = 10. Values are in Mean ± SD *Significantly difference vs Control group (P < 0.05, Student’s t test).

GLU: Glucose, CRE: Creatinine, TCHO: Total Cholesterol, TRIG: Triglycerides, ALT: Alanine Aminotransferase, AST: Aspartate Aminotransferase, TP: Total protein, HDL: High Density Lipoprotein, LDL: Low Density Lipoprotein, ALB: Albumin, GGT: Gamma Glutamyl Transpeptidase, CA: Calcium, PHO: Phosphate, GLOB: Globulin, TBA: Total bile acids, ALP: Alkaline phosphatase, TBIL: Total Bilirubin, BUN: Blood Urea Nitrogen, NA: Sodium, K: Potassium, CL: Chloride

**Table 7:** Summary of the recovery male and female Sprague-Dawley rats clinical chemistry parameters after 90 days of oral repeated dose of SCFE-50 C.

	Recovery males		Recovery females	
	Control	SCFE-50 C (mg/kg BW/day)	Control	SCFE-50 C (mg/kg BW/day)
		2000		2000
GLU (mg/dL)	103.62 ± 16.02	96.06 ± 20.72	101.11 ± 29.16	100.54 ± 26.9
UREA (mg/dL)	34.59 ± 4.16	33.27 ± 4.09	31.15 ± 9.53	31.36 ± 8.26
CRE (mg/dL)	0.77 ± 0.02	0.71 * ± 0.03	0.67 ± 0.24	0.76 ± 0.2
TCHO (mg/dL)	49.33 ± 7.6	48.66 ± 11.22	54.71 ± 20.85	60.36 ± 19.19
TRIG (mg/dL)	56.97 ± 25.32	51.67 ± 14.16	51.64 ± 23.17	52.67 ± 20.41
AST (U/L)	145.86 ± 7.86	162.03 ± 22.33	132.12 ± 40.86	147.98 ± 50.94
ALT (U/L)	51.28 ± 11.37	52.56 ± 4.95	35.88 ± 14.85	44.34 ± 17.15
TP (g/L)	68.76 ± 0.95	67.71 ± 1.18	67.52 ± 23.12	68.46 * ± 16.57
HDL (mg/dL)	15.67 ± 0.66	14.28 ± 2.5	25.49 ± 10.65	26.99 ± 8.97
LDL (mg/dL)	12.94 ± 3.55	12.48 ± 5.31	18.18 ± 9.17	23.25 ± 9.07
ALB (g/L)	24.66 ± 8.92	21.51 ± 6.28	24.93 ± 10.06	22.94 * ± 8.51
GGT (U/L)	-1.31 ± 1.3	-4.24 ± 3.42	-1.83 ± 6.05	-0.24 ± 2.67
CA (mmol/L)	3.26 ± 0.39	2.98 ± 0.51	2.21 ± 0.86	2.52 ± 0.9
PHO (mg/dL)	9.22 ± 1.38	9.59 ± 4.13	5.43 ± 2.65	4.55 ± 2.14
GLOB (g/L)	44.1 ± 8.11	46.2 ± 6.16	44.42 ± 14.87	45.88 * ± 14.27
TBA (µmol/L)	22.75 ± 11.8	24.77 ± 7.82	42.28 ± 43.41	49.08 ± 40.4
ALP (U/L)	63.09 ± 26.71	56.38 ± 14.29	46.78 ± 23.42	35.65 ± 24.3
TBIL (mg/dL)	0.08 ± 0.12	0.07 ± 0.06	0.1 ± 0.16	0.01 ± 0.07
BUN (mg/dL)	16.16 ± 1.94	15.54 ± 1.91	14.55 ± 4.45	14.65 ± 3.86
NA (mmol/L)	144.2 ± 2.59	144 ± 1	130.82 ± 45.37	132.42 * ± 30.89
K (mmol/L)	4.94 ± 0.36	4.8 ± 0.2	4.3 ± 1.47	4.23 ± 1.09
CL (mmol/L)	104.6 ± 1.52	103.00 * ± 0.71	95.27 ± 32.87	95.71 * ± 22.28

N = 5. Values are in Mean ± SD *Significantly difference vs Control group (P < 0.05, Student’s t test).

GLU: Glucose, CRE: Creatinine, TCHO: Total Cholesterol, TRIG: Triglycerides, ALT: Alanine Aminotransferase, AST: Aspartate Aminotransferase, TP: Total protein, HDL: High Density Lipoprotein, LDL: Low Density Lipoprotein, ALB: Albumin, GGT: Gamma Glutamyl Transpeptidase, CA: Calcium, PHO: Phosphate, GLOB: Globulin, TBA: Total bile acids, ALP: Alkaline phosphatase, TBIL: Total Bilirubin, BUN: Blood Urea Nitrogen, NA: Sodium, K: Potassium, CL: Chloride

### 3.7 Serum thyroid hormone estimation

There were no SCFE-50 C related changes in the serum hormone levels in any of the group. In high dose female group (G4), there was a significant decrease in the level of T3 (505.1 pg/mL) and decreased TSH (1.2 µIU/mL) compared with vehicle control group G1 (T3 = 582.3 pg/dL, T4 = 1.4 µIU/mL) observed. However, no corresponding changes were observed during the histopathology examination. In recovery group male rats, a statistically significant decrease in the level of T4 (25.1 ng/mL) (G1R= 28.4 ng/mL) was observed and it was considered as incidental as no change in the level of TSH and T4 were observed and also no change in the level of T4 was observed at the end of the treatment period. The summarized values for male and female rats are shown in Table 8 and for recovery male and female rats are shown Table 9.

**Table 8:** Summary of the male and female Sprague-Dawley rats thyroid hormone estimation after 90 days of oral repeated dose of SCFE-50 C.

	Males				Females			
	Control	SCFE-50 C (mg/kg BW/day)			Control	SCFE-50 C (mg/kg BW/day)		
Parameter		500	1000	2000		500	1000	2000
T3 (pg/mL)	571.0119 ± 83.3685	602.9509 ± 85.2566	504.8818 ± 82.9976	523.9223 ± 51.038	582.2724 ± 52.0031	603.9745 ± 102.2183	538.6634 ± 69.8741	505.0865 * ± 34.3954
T4 (ng/mL)	25.3177 ± 2.9577	25.3715 ± 2.1354	26.0833 ± 2.0289	25.5192 ± 2.4669	26.3116 ± 2.8865	25.17 ± 5.0976	24.7805 ± 3.12	23.4105 ± 3.3843
TSH (µIU/mL)	1.3165 ± 0.3498	1.3411 ± 0.2587	1.3999 ± 0.2161	1.177 ± 0.1831	1.4388 ± 0.1644	1.4382 ± 0.3307	1.4205 ± 0.2585	1.1513 * ± 0.1224

N = 10. Values are in Mean ± SD *Significantly difference vs Control group (P < 0.05, Student’s t test).

T3: Triiodothyronine, T4: Thyroxine, TSH: Thyroxine stimulating Hormone

**Table 9:** Summary of the recovery male and female Sprague-Dawley rats thyroid hormone estimation after 90 days of oral repeated dose of SCFE-50 C.

	Recovery Males		Recovery females	
	Control	SCFE-50 C (mg/kg BW/day)	Control	SCFE-50 C (mg/kg BW/day)
Parameter		2000		2000
T3 (pg/mL)	557.7039 ± 81.5049	585.5482 ± 60.4928	626.0861 ± 39.2861	581.8629 ± 91.0263
T4 (ng/mL)	28.4203 ± 2.7355	25.1431 * ± 0.7258	25.5998 ± 1.8479	24.3641 ± 3.8456
TSH (µIU/mL)	1.3925 ± 0.4468	1.2336 ± 0.2487	1.4085 ± 0.169	1.4188 ± 0.1808

N = 5. Values are in Mean ± SD *Significantly difference vs Control group (P < 0.05, Student’s t test).

T3: Triiodothyronine, T4: Thyroxine, TSH: Thyroxine stimulating Hormone

### 3.8 Urinalysis observation

There were no statistically significant changes in the urine volume in any Capsanthin treated groups and control groups except the recovery group. The urine output volume was found to be increased in both recovery male (14 mL) (G1R= 10 mL) and female group (15 mL) (G1R= 10 mL) in comparison to the control group animals. Besides, the incidental occurrence of WBC, brown colour urine, and protein in urine was observed in a few males and females of all groups including control. These findings were considered as incidental as there were no major histopathological changes in the kidney and lower urinary tract. The summarized values for male and female rats are presented in Table 10 and for recovery male and female groups are shown in Table 11.

**Table 10:** Summary of the male and female Sprague-Dawley rats urinalysis parameters after 90 days of oral repeated dose of SCFE-50 C.

	Males				Females			
	Control	SCFE-50 C (mg/kg BW/day)			Control	SCFE-50 C (mg/kg BW/day)		
Parameter		500	1000	2000		500	1000	2000
APP^	0/10	0/10	0/10	0/10	4/10	5/10	4/10	1/10
VOL (mL)	16 ± 8	13 ± 4	12 ± 4	19 ± 9	5 ± 1	6 ± 2	9 ± 4	9 ± 7
SPG	1.019 ± 0.006	1.02 ± 0.005	1.018 ± 0.005	1.019 ± 0.007	1.021 ± 0.004	1.02 ± 0.006	1.022 ± 0.004	1.02 ± 0.006
pH	8.8 ± 0.4	8.8 ± 0.4	8.7 ± 0.5	8.6 ± 0.5	9 ± 1	8 ± 1	8 ± 1	8 ± 1
WBC^ (No/µL)	1/10	0/10	0/10	1/10	1/10	0/10	2/10	2/10
PRO^ (mg/dL)	0/10	0/10	1/10	0/10	2/10	1/10	0/10	2/10
GLU^ (mg/dL)	0/10	0/10	0/10	0/10	0/10	0/10	0/10	0/10
ERY^ (No/µL)	0/10	0/10	0/10	0/10	0/10	0/10	0/10	0/10

^ Incidents of finding

N = 10. Values are in Mean ± SD. No significant difference between treated and Control group (Student’s t test).

APP: Appearance, VOL: Volume, SPG: Specific gravity, WBC: Total leukocyte count, PRO: Protein, GLU: Glucose, ERY: Erythrocytes

**Table 11:** Summary of the recovery male and female Sprague-Dawley rats urinalysis parameters after 90 days of oral repeated dose of SCFE-50 C.

	Recovery males		Recovery females	
	Control	SCFE-50 C (mg/kg BW/day)	Control	SCFE-50 C (mg/kg BW/day)
Parameter		2000		2000
APP^	0/5	0/5	0/5	0/5
VOL (mL)	10 ± 1	14 * ± 412 ± 419 ± 9	10 ± 2	15 * ± 39 ± 49 ± 7
SPG	1.018 ± 0.004	1.019 ± 0.007	1.021 ± 0.002	1.020 ± 0.004
pH	8.0 ± 0.7	8.6 ± 0.5	8 ± 5	8 ± 1
WBC^ (No/µL)	0/5	1/5	0/5	0/5
PRO^ (mg/dL)	0/5	0/5	0/5	0/5
GLU^ (mg/dL)	0/5	0/5	0/5	0/5
ERY^ (No/µL)	0/5	0/5	0/5	0/5

^ Incidents of finding

N = 5. Values are in Mean ± SD *Significantly difference vs Control group (P < 0.05, Student’s t test).

APP: Appearance, VOL: Volume, SPG: Specific gravity, WBC: Total leukocyte count, PRO: Protein, GLU: Glucose, ERY: Erythrocytes

### 3.9 Organ weights, necropsy, and gross pathology findings

There were no significant changes in either absolute body weight or relative organ weight in the treated as well as control groups. Male animals in the main group showed a statistically significant decrease in absolute thyroid gland weight (G2–0.16 g, G4–0.17 g) when compared to the control group (G1–0.18 g). Also, there was an increase in brain weight in the recovery male group (2.28 g) when compared to the control group animals (2.08 g). These changes were considered as incidental as there were no supporting gross and microscopic changes. For male and female groups, body weight, the absolute and relative organ weights are shown in Table 12. For male and female recovery groups, body weight, the absolute and relative organ weights are shown in Table 13.

**Table 12:** Summary of the male and female Sprague-Dawley rats body weight, absolute and relative organ weights after 90 days of oral repeated dose of SCFE-50 C.

	Males							Females					
		SCFE-50 C (mg/kg BW/day)								SCFE-50 C (mg/kg BW/day)			
	Control	500		1000		2000		Control		500	1000		2000
Absolute organ weights													
Body weight	517.86 ± 48.13	511.4 ± 49.20		518.58 ± 59.47		527.6 ± 62.62		293.36 ± 22.27		285.88 ± 23.73	282.82 ± 24.88		299.44 ± 26.71
Liver	13.51 ± 2.27	12.26 ± 1.71		13.63 ± 3.76		14.19 ± 3.11		8.52 ± 0.85		8.2 ± 1.11	7.9 ± 0.87		8.37 ± 0.91
Kidneys	3.21 ± 0.33	2.89 ± 0.36		3.2 ± 0.59		3.01 ± 0.39		1.86 ± 0.22		1.75 ± 0.20	1.78 ± 0.20		1.79 ± 0.18
Adrenals	0.07 ± 0.02	0.07 ± 0.01		0.06 ± 0.02		0.07 ± 0.02		0.07 ± 0.01		0.07 ± 0.02	0.08 ± 0.01		0.07 ± 0.02
Spleen	0.8 ± 0.10	0.73 ± 0.14		0.79 ± 0.19		0.77 ± 0.1		0.53 ± 0.10		0.47 ± 0.08	0.55 ± 0.08		0.5 ± 0.04
Heart	1.79 ± 0.29	1.65 ± 0.30		1.71 ± 0.30		1.72 ± 0.27		1.06 ± 0.14		0.99 ± 0.07	1.09 ± 0.12		1.07 ± 0.08
Thymus	0.39 ± 0.10	0.41 ± 0.06		0.42 ± 0.07		0.47 ± 0.09		0.38 ± 0.09		0.31 ± 0.09	0.36 ± 0.09		0.34 ± 0.08
Brain	2.23 ± 0.16	2.23 ± 0.12		2.13 ± 0.17		2.11 ± 0.16		2.04 ± 0.06		2 ± 0.13	2.04 ± 0.14		1.92 ± 0.15
Thyroid / Parathyroid gland	0.18 ± 0.01	0.16 * ± 0.00		0.18 ± 0.00		0.17 * ± 0.00		0.17 ± 0.01		0.17 ± 0.01	0.17 ± 0.00		0.17 ± 0.00
Pituitary gland	0.02 ± 0.00	0.02 ± 0.00		0.02 ± 0.00		0.02 ± 0.00		0.02 ± 0.00		0.02 ± 0.00	0.02 ± 0.00		0.02 ± 0.00
Prostate +seminal vesicles with coagulating glands	2.5 ± 0.67	2.78 ± 0.95		2.94 ± 0.93		2.24 ± 0.6		–		–	–		–
Testes	3.74 ± 0.22	3.6 ± 0.29		3.65 ± 0.35		3.48 ± 0.22		–		–	–		–
Epididymis	1.55 ± 0.11	1.63 ± 0.15		1.74 ± 0.45		1.57 ± 0.29							
Ovaries	–	–		–		–		0.2 ± 0.06		0.19 ± 0.03	0.2 ± 0.05		0.22 ± 0.02
Uterus	–	–		–		–		0.76 ± 0.05		0.75 ± 0.14	0.73 ± 0.20		0.82 ± 0.25
Relative organ weights													
Liver	2.6 ± 0.28		2.4 ± 0.21		2.6 ± 0.50		2.67 ± 0.36	1.65 ± 0.15	1.61 ± 0.22			1.53 ± 0.50	1.6 ± 0.22
Kidneys	0.62 ± 0.05		0.56 ± 0.04		0.61 ± 0.06		0.57 ± 0.04	0.36 ± 0.04	0.34 ± 0.03			0.35 ± 0.06	0.34 ± 0.05
Adrenals	0.01 ± 0.00		0.01 ± 0.00		0.01 ± 0.00		0.01 ± 0.01	0.01 ± 0.00	0.01 ± 0.00			0.01 ± 0.00	0.01 ± 0.00
Spleen	0.15 ± 0.01		0.14 ± 0.03		0.15 ± 0.03		0.15 ± 0.01	0.1 ± 0.01	0.09 ± 0.01			0.11 ± 0.03	0.1 ± 0.01
Heart	0.35 ± 0.04		0.32 ± 0.06		0.33 ± 0.05		0.33 ± 0.06	0.21 ± 0.02	0.2 ± 0.02			0.21 ± 0.05	0.21 ± 0.03
Thymus	0.08 ± 0.02		0.08 ± 0.01		0.08 ± 0.01		0.09 ± 0.02	0.07 ± 0.02	0.06 ± 0.02			0.07 ± 0.01	0.06 ± 0.02
Brain	0.43 ± 0.04		0.44 ± 0.05		0.41 ± 0.04		0.41 ± 0.07	0.4 ± 0.04	0.39 ± 0.03			0.4 ± 0.04	0.37 ± 0.05
Thyroid / Parathyroid gland	0.03 ± 0.00		0.03 ± 0.00		0.03 ± 0.00		0.03 ± 0.00	0.03 ± 0.00	0.03 ± 0.00			0.03 ± 0.00	0.03 ± 0.00
Pituitary gland	0 ± 0.00		0.003 ± 0.001		0.004 ± 0.001		0.004 ± 0.001	0.004 ± 0.001	0.003 ± 0.001			0.003 ± 0.001	0.004 ± 0.001
Prostate +seminal vesicles with coagulating glands	0.48 ± 0.12		0.54 ± 0.18		0.56 ± 0.16		0.43 ± 0.10	–	–			–	–
Testes	0.73 ± 0.10		0.71 ± 0.10		0.71 ± 0.08		0.67 ± 0.07	–	–			–	–
Epididymis	0.3 ± 0.03		0.32 ± 0.05		0.33 ± 0.07		0.3 ± 0.07						
Ovaries	–		–		–			0.04 ± 0.01	0.04 ± 0.00			0.04 ± 0.07	0.04 ± 0.01
Uterus	–		–		–		–	0.15 ± 0.03	0.15 ± 0.04			0.16 ± 0.16	0.15 ± 0.04

N = 10. Values are in Mean ± SD *Significantly difference vs Control group (P < 0.05, Student’s t test).

**Table 13:** Summary of the recovery male and female Sprague-Dawley rats body weight, absolute and relative organ weights after 90 days of oral repeated dose of SCFE-50 C.

	Recovery male		Recovery female	
		SCFE-50 C (mg/kg BW/day)		SCFE-50 C (mg/kg BW/day)
	Control	2000	Control	2000
Absolute organ weights				
**Body weight**	550.23 ± 55.62	589.32 ± 102.87	292.46 ± 15.50	285.88 ± 23.73
**Liver**	14.73 ± 2.09	16.82 ± 4.06	8.52 ± 1.40	9.15 ± 1.27
**Kidneys**	3.47 ± 0.47	3.62 ± 0.56	1.84 ± 0.13	1.98 ± 0.12
**Adrenals**	0.06 ± 0.02	0.06 ± 0.01	0.06 ± 0.03	0.06 ± 0.02
**Spleen**	0.75 ± 0.11	0.88 ± 0.25	0.5 ± 0.07	0.47 ± 0.10
**Heart**	1.84 ± 0.25	1.75 ± 0.23	1.02 ± 0.09	1.11 ± 0.07
**Thymus**	0.35 ± 0.08	0.44 ± 0.13	0.26 ± 0.09	0.3 ± 0.06
**Brain**	2.08 ± 0.21	2.28 * ± 0.13	1.89 ± 0.11	1.9 ± 0.12
**Thyroid / Parathyroid gland**	3.57 ± 0.46	3.54 ± 0.09	0.28 ± 0.08	0.33 ± 0.09
**Pituitary gland**	1.66 ± 0.19	1.72 ± 0.24	0.02 ± 0.00	0.01 ± 0.00
**Prostate +seminal vesicles with coagulating glands**	3.05 ± 0.75	2.86 ± 0.19	–	–
**Testes**	0.4 ± 0.16	0.38 ± 0.13	–	–
**Epididymis**	0.02 ± 0.01	0.01 ± 0.00	–	–
**Ovaries**	–	–	0.18 ± 0.04	0.17 ± 0.04
**Uterus**	–	–	0.9 ± 0.34	0.8 ± 0.12
Relative organ weights				
**Liver**	2.7 ± 0.33	2.84 ± 0.31	1.56 ± 0.21	1.57 ± 0.25
**Kidneys**	0.62 ± 0.06	0.62 ± 0.04	0.34 ± 0.03	0.34 ± 0.04
**Adrenals**	0.01 ± 0.00	0.01 ± 0.00	0.01 ± 0.00	0.01 ± 0.01
**Spleen**	0.14 ± 0.02	0.15 ± 0.02	0.09 ± 0.01	0.08 ± 0.01
**Heart**	0.34 ± 0.03	0.3 ± 0.02	0.19 ± 0.02	0.19 ± 0.03
**Thymus**	0.06 ± 0.01	0.07 ± 0.02	0.05 ± 0.02	0.05 ± 0.01
**Brain**	0.38 ± 0.07	0.39 ± 0.04	0.35 ± 0.03	0.33 ± 0.07
**Thyroid / Parathyroid gland**	0.65 ± 0.07	0.61 ± 0.09	0.05 ± 0.01	0.06 ± 0.02
**Pituitary gland**	0.3 ± 0.02	0.3 ± 0.06	0.003 ± 0.000	0.002 ± 0.000
**Prostate +seminal vesicles with coagulating glands**	0.55 ± 0.12	0.49 ± 0.07	–	–
**Testes**	0.07 ± 0.03	0.07 ± 0.03	–	–
**Epididymis**	0.004 ± 0.002	0.002 ± 0.001	–	–
**Ovaries**	–	–	0.03 ± 0.01	0.03 ± 0.01
**Uterus**	–	–	0.17 ± 0.07	0.14 ± 0.02

N = 5. Values are in Mean ± SD *Significantly difference vs Control group (P < 0.05, Student’s t test).

During necropsy, all the animals were observed for any external or internal abnormality. Gross external and internal abnormality were not observed in any of the group. External examination revealed mild focal alopecia in a few animals and is considered it to be an incidental change. However, there were no gross pathological changes in visceral organs. Besides, the cell population was found to be normal across the groups during the vaginal cytology examination in females. No variation was observed in organ weights and histological details of all female reproductive organs.

### 3.10 Histopathology

Histopathological examination of preserved organs collected from the vehicle control (G1) and high dose groups (G4) was examined to know the effect of the test item on these organs. Since there were no test item-related changes noted in any of the organs in the high dose group, an examination of organs from the low dose group was not carried out. Lungs showed thickening of the interstitium and peribronchiolar cell infiltration in a few animals from both control and high dose group with mild severity.

Only one male and female animal each from the high dose group showed moderate and severe interstitial thickening. Since the incidence of occurrence of these changes was comparable to the control group and it was considered as an incidental finding rather than test item induced. The Liver showed minimal to mild severity of hepatocellular degeneration and infiltration of inflammatory cells in both high dose and control group animals. This low severity degenerative process may be reversible as there were no signs of necrosis of hepatocytes. Further, the incidence of occurrence of degenerative changes was comparable to the vehicle control group. Also, there were no visible gross lesions in the liver and no variations in the absolute weight of the liver at the end of the recovery study period.

Kidney revealed tubular degenerative changes in a few animals from both the control and high dose groups. Administration of corn oil at the usual dose rate of 10 mL/kg as a vehicle of a test agent to rats resulted in toxic effects on the kidney. Histopathologic findings of corn oil 10 mL/kg fed male rats showed severe epithelial necrosis and fatty degeneration of the proximal tubule and female rats showed severe fatty degeneration of proximal tubules tended to have both necrosis and basophilic tubules [27]. In this study, the tubular degenerative changes in both control and treated group may be attributed to corn oil treatment. The summary of histopathology details for male and female rats for the groups G1 and G4 is shown in Table 14.

**Table 14:** Summary of the male and female Sprague-Dawley rats histopathology observation after 90 days of oral repeated dose of SCFE-50 C.

Tissue with lesion	Male		Female		
	Control		SCFE-50 C (mg/kg BW/day)	Control	SCFE-50 C (mg/kg BW/day)
			2000		2000
Adrenal glands	0/10		0/10	0/10	0/10
Aorta	0/10		0/10	0/10	0/10
Axillary/neck lymph node	0/10		0/10	0/10	0/10
Bone and bone marrow (femur)	0/10		0/10	0/10	0/10
Brain (cerebrum, cerebellum, medulla/pons)	0/10		0/10	0/10	0/10
Eyes (with optic nerve)	0/10		0/10	0/10	0/10
Heart	1/10		0/10	1/10	0/10
Gastrointestinal tract					
-Cecum	0/10		0/10	0/10	0/10
-Colon	0/10		0/10	0/10	0/10
-Duodenum	0/10		0/10	0/10	0/10
-Epididymites	0/10		0/10	0/10	0/10
-Esophagus	0/10		0/10	0/10	0/10
-Ileum with Peyer’s Patch- within normal limit	0/10		0/10	0/10	0/10
-Jejunum- within normal limit	0/10		0/10	0/10	0/10
Kidneys	4/10		5/10	3/10	3/10
-Interstitial hemorrhage - minimal multifocal	0/10		2/10	0/10	0/10
-Interstitial hemorrhage - mild multifocal	1/10		2/10	1/10	1/10
-Tubular degeneration-minimal multifocal	2/10		1/10	1/10	0/10
-Tubular degeneration - mild diffuse	1/10		1/10	1/10	2/10 (1.0)
-Infiltration of Inflammatory cell - minimal multifocal	0/10		0/10	0/10	0/10
Liver	4/10		6/10	5/10	6/10
-Infiltration of inflammatory cell - minimal multifocal	0/10		1/10	0/10	1/10
-Infiltration of inflammatory cell - mild multifocal	0/10		1/10	0/10	1/10
-Hepatocellular degeneration - mild multifocal	1/10		0/10	1/10	1/10
-Hepatocellular degeneration - minimal diffuse	0/10		1/10	0/10	1/10
-Hepatocellular degeneration - mild diffuse	2/10		2/10	2/10	1/10
-Sinusoidal congestion - mild diffuse	2/10		2/10	2/10	3/10
Lungs	3/10		4/10	6/10	4/10
-Thickening of interstitium - mild multifocal	2/10		0/10	0/10	3/10 (1.0)
-Thickening of interstitium - mild diffuse	1/10		2/10	4/10	0/10
-Thickening of interstitium - moderate diffuse	0/10		1/10	0/10	0/10
-Thickening of interstitium - severe diffuse	0/10		0/10	0/10	1/10
-Peribronchiolar cell infiltration - mild multifocal	1/10		2/10	2/10	0/10
Mesenteric lymph nodes	0/10	0/10	0/10		0/10
Skin (with mammary gland for male and female)	0/10	0/10	0/10		0/10
Skeletal muscle	0/10	0/10	0/10		0/10
Nerve, sciatic	0/10	0/10	0/10		0/10
Pituitary gland	0/10	0/10	0/10		0/10
Salivary glands	0/10	0/10	0/10		0/10
Spinal cord (cervical, thoracic and lumbar)	0/10	0/10	0/10		0/10
Thymus	0/10	0/10	0/10		0/10
Thyroid with parathyroids	0/10	0/10	0/10		0/10
Trachea	0/10	0/10	0/10		0/10
Lymphoid depletion-minimal multifocal	0/10	1/10	0/10		0/10
Stomach-	0/10	0/10	0/10		0/10
Pancreas	0/10	0/10	0/10		0/10
Spleen	0/10	1/10	0/10		0/10
Testes	0/10	0/10	0/10		0/10
Prostate + seminal vesicles with coagulating glands	0/10	0/10	0/10		0/10
Vagina	0/10	0/10	0/10		0/10
Urinary bladder	0/10	0/10	0/10		0/10
Uterus with cervix	0/10	0/10	0/10		0/10
Ovaries	0/10	0/10	0/10		0/10
Rectum	0/10	0/10	0/10		0/10

The values shown are the number affected/number evaluated (mean severity of affected animals).

The severity score shown is the highest recorded. Severity scores are out of a grade of 0 (no injury), 1 (minimal), 2 (mild), 3 (moderate), 4 (severe), and 5 (extreme)

### 3.11 Capsanthin levels in plasma and macula

In positive ESI mode, the presence of capsanthin was confirmed using the *m/z* value of 585.51. In plasma, capsanthin was not detected in the vehicle control group. In the treated groups, the capsanthin in low (G2), mid (G3) and high dose (G4) groups were 0.46 ng/mL, 0.93 ng/mL, and 1.8 ng/mL respectively. Dose-dependent increase level was seen in plasma. However, capsanthin was not detected in the macula of the Vehicle control (G1) and low dose (G2) and mid-dose (G3) groups but in the high dose group (G4) the capsanthin content was 0.38 ng/mL. The cumulative data for the capsanthin presence in plasma and eye macula for the groups G1, G2, G3, and G4 are shown in Table 15.

**Table 15:** Summary of the presence of capsanthin in plasma and macula of Sprague-Dawley rats after 90 days of oral repeated dose of SCFE-50 C.

		Day 90										
		SCFE-50 C (mg/kg BW/day)										
		Control			500			1000			2000	
Capsanthin concentration	ng/mL	Plasma	Macula	Plasma		Macula	Plasma		Macula	Plasma		Macula
		ND	ND	0.46		ND	0.93		ND	1.8		0.38

ND: Not detected

Waters LCMS/MS equipped with MassLynx V4.1 software. Instrument conditions-mode: positive ion spray, spray source: quadrupole time of flight (QTof), desolvation gas flow: 800 L/hr., desolvation temperature: 450ºC, source temperature: 120ºC, capillary voltage: 2.5KV, Cone:40 V, Cone gas flow: 50 L/hr. Isocratic mobile phase- acetonitrile and 5 mM ammonium acetate (80:20 v/v) with 0.1% formic acid. Flow rate − 0.7 mL/min. Column-Atlantis®T3 column. Dimension-4.6 × 75 mm and particle size 5 µm. The retention time for capsanthin in reference standard, capsanthin in plasma and capsanthin in macula were 7.10, 7.13 and 7.09 min respectively. The presence of capsanthin was confirmed using the *m/z* value of 585.51.

## 4 Discussion

Of late, researchers are exploring the potential biological activities of certain bioactive molecules found in commonly consumed fruits and vegetables on human wellbeing. *Capsicum annum* ethyl acetate extract protects the rat brain from rotenone-induced neurotoxicity through regulating dopamine metabolism and GSH redox [28]. Carotenoids from *Capsicum annum* are a few of the foremost chemically and functionally distinct molecules in food [29]. The unique biological properties of carotenoids such as radical scavenging, positive effect on inflammatory markers, and preventive effect of neurodegenerative disorders are due to their unique structure. The defensive role of carotenoids especially in the prevention of age-related macular degeneration [30] is well established. Capsanthin and capsorubin are the most potential antioxidant carotenoids of red bell pepper and responsible for red color [31]. While various therapeutic benefits have been reported for capsanthin, few toxicity studies were reported. The acute oral toxicity of red pepper color in rats was reported with an LD_50_ exceeding 11.25 g/kg BW [32]. In a 13-week rat acute toxicity study of capsicum color preparation extracted from Spanish paprika fruit with hexane on F344/DuCrj rats [33], no significant changes in general characteristics, no mortality, no change in organ weights, and no histopathological changes were observed in any experimental groups.

SCFE-50 C, a novel, first-ever red bell pepper extract enriched to capsanthin > 50% (w/w) was evaluated to confirm its toxicity in rats. No abnormalities and mortalities were observed in any group throughout the study. No change in general behavior, clinical biochemistry, hematology, urinary examination in the treated group compared to control. Analysis of serum thyroid hormone, necropsy, and histopathology examination did not reveal any statistically significant adverse events in SCFE-50 C treated groups. There were statistically significant changes in some parameters in SCFE-50 C treated groups compared to the control as given below but the clinical signs only appear during the treatment, recovers when the SCFE-50 C treatment was stopped. In male rats, a statistically significant lower body weight gain in G2, higher body weight gain in G3 were observed. In female rats, lower body weight gain in G2 were observed (Fig. 3). In male rats, a statistically significant increase in the eosinophil count in G2 and G3 when compared with the control group.

The weekly percentage mean body weight change of the vehicle control and treated groups on week six was lower (Fig. 2 & 3) and over the course of the experiment was within the acceptable range.

Females showed a statistically significant decrease in WBC count in G4 and an increase in the level of platelet in G2, G3 and G4 groups compared to control group (Table 4). A statistically significant increase in the level of creatinine was observed in G3 male rats whereas the glucose level was increased in G2 female rats when compared to control group (Table 5). In G4 female rats, a significant decrease in T3 and TSH was observed (Table 8). In both control and high dose treated groups, mild severity of thickening of interstitium in lungs, hepatocellular degeneration in the liver, and tubular degenerative changes in the kidney were observed (Table 14). These changes observed were not consistent and not to be considered toxicologically significant because they are not dose-dependent and returned to normal at the end of the study period.

In summary, the findings from present animal toxicity study support the conclusion that SCFE-50 C is unlikely to cause adverse effects in SD rats. Based on the results of the 90-day study, the no-observed-effect-level (NOAEL) of saponified *Capsicum annum* fruit extract with 50% (w/w) capsanthin (SCFE-50 C) was found to be more than 2000 mg/kg BW/day, the highest dose tested.

## Declaration of Competing Interest

Velmurugan Shanmugham and Ravi Subban are associated with Karpagam Academy of Higher Education, Coimbatore, India. They have been sponsored by Unibar Corporation, USA to conduct the study at the Vipragen Biosciences, India.
